# The Krüppel-like factor 9 (KLF9) network in HEC-1-A endometrial carcinoma cells suggests the carcinogenic potential of dys-regulated KLF9 expression

**DOI:** 10.1186/1477-7827-6-41

**Published:** 2008-09-10

**Authors:** Frank A Simmen, Ying Su, Rijin Xiao, Zhaoyang Zeng, Rosalia CM Simmen

**Affiliations:** 1 Department of Physiology and Biophysics, and the Arkansas Children's Nutrition Center, 1212 Marshall Street, University of Arkansas for Medical Sciences, Little Rock, Arkansas, 72202, USA

## Abstract

**Background:**

Krüppel-like factor 9 (KLF9) is a transcriptional regulator of uterine endometrial cell proliferation, adhesion and differentiation; processes essential for pregnancy success and which are subverted during tumorigenesis. The network of endometrial genes controlled by KLF9 is largely unknown. Over-expression of KLF9 in the human endometrial cancer cell line HEC-1-A alters cell morphology, proliferative indices, and differentiation, when compared to KLF9 under-expressing HEC-1-A cells. This cell line provides a unique model for identifying KLF9 downstream gene targets and signaling pathways.

**Methods:**

HEC-1-A sub-lines differing in relative levels of KLF9 were subjected to microarray analysis to identify differentially-regulated RNAs.

**Results:**

KLF9 under-expression induced twenty four genes. The KLF9-suppressed mRNAs encode protein participants in: aldehyde metabolism (AKR7A2, ALDH1A1); regulation of the actin cytoskeleton and cell motility (e.g., ANK3, ITGB8); cellular detoxification (SULT1A1, ABCC4); cellular signaling (e.g., ACBD3, FZD5, RAB25, CALB1); and transcriptional regulation (PAX2, STAT1). Sixty mRNAs were more abundant in KLF9 over-expressing sub-lines. The KLF9-induced mRNAs encode proteins which participate in: regulation and function of the actin cytoskeleton (COTL1, FSCN1, FXYD5, MYO10); cell adhesion, extracellular matrix and basement membrane formation (e.g., AMIGO2, COL4A1, COL4A2, LAMC2, NID2); transport (CLIC4); cellular signaling (e.g., BCAR3, MAPKAPK3); transcriptional regulation [e.g., KLF4, NR3C1 (glucocorticoid receptor), RXRα], growth factor/cytokine actions (SLPI, BDNF); and membrane-associated proteins and receptors (e.g., CXCR4, PTCH1). In addition, the abundance of mRNAs that encode hypothetical proteins (KLF9-inhibited: C12orf29 and C1orf186; KLF9-induced: C10orf38 and C9orf167) were altered by KLF9 expression. Human endometrial tumors of high tumor grade had decreased KLF9 mRNA abundance.

**Conclusion:**

KLF9 influences the expression of uterine epithelial genes through mechanisms likely involving its transcriptional activator and repressor functions and which may underlie altered tumor biology with aberrant KLF9 expression.

## Background

Krüppel-like factor 9 (KLF9), previously referred to as Basic Transcription Element Binding (BTEB) Protein 1, is an evolutionarily well-conserved member of the Krüppel-like factor (KLF) family of transcriptional regulators, so named for the presence of *Drosophila *Krüppel-like DNA-binding domain(s) in the protein's C-terminal region [1]. KLFs function as transcriptional activators or repressors, depending upon cellular context and presence of partner co-regulators [1,2]. Individual KLF proteins affect cell proliferation, differentiation, apoptosis, DNA damage, and stress responses [2]. Members of this family have been implicated in stem cell renewal, maintenance of pluripotency, lineage determination, organogenesis, and oncogenesis [2-5], underscoring their wide-ranging regulatory roles in development.

KLF9 was first isolated and identified as trans-repressor of the rat liver CYP1A1 gene and inducer of SV40 early and HIV-1 long terminal repeat promoters [6,7]. Subsequent work demonstrated KLF9 activation of the liver CYP7A gene [8]. Albeit trans-activation and trans-repression functions of KLF9 are mediated by formation of different transcriptional protein complexes, they occur through KLF9 binding to highly similar GC/GT boxes [2,6,7]. KLF9 mRNA is expressed at highest levels in rat brain, kidney, lung and testis [6]. Potential brain functions for KLF9 have been recently elucidated. This transcription factor is induced in rat brain by 3, 5, 3'-triiodothyronine (T_3_) and mediates effects of T_3 _on neuronal process development [9-11]. Mice lacking KLF9 exhibited deficits in motor learning, motor coordination and fear-conditioning [12]. Recently, KLF9 was shown to regulate crypt-villus cell renewal in mice [13] and secondary antibody responses of human splenic B cells [14].

The uterus is a complex organ that exhibits hormonally (estrogen, progesterone) driven changes in the expression of myriad genes and gene products during the estrous cycle and pregnancy. Morphologically, the uterus is comprised of epithelium (glandular and luminal), stromal fibroblasts, immune cells, and myometrium. Regulatory actions of KLF9 in the uterus during development and pregnancy have been partially elucidated by our laboratories. Mice null for KLF9 exhibited uterine hypoplasia, smaller litter size, reduced numbers of implantation sites, partial progesterone resistance in the uterus, and delayed parturition [15,16]. The sub-fertility phenotype of the KLF9 knockout mouse was shown to correlate with aberrant timing of uterine stromal and epithelial proliferation during the peri-implantation period, suggesting an out-of-phase uterus relative to blastocyst development as potentially causative for sub-fertility of KLF9 null females [17]. KLF9 facilitated progesterone-inductive effects on uterine gene expression by its co-recruitment with the progesterone receptor [18,19] and inhibited estrogen receptor α trans-activity by promoting this receptor's estrogen-induced down-regulation [20].

In previous studies, analysis of estrogen receptor-negative human endometrial carcinoma (HEC-1-A) cells that were genetically engineered to over- or under-express KLF9 identified serum-dependent mitogenic functions for this nuclear protein, based on its influence on cell phenotype and gene expression changes that support increased proliferation [21,22]. HEC-1-A cells with increased KLF9 expression grew as flat monolayers, whereas those with less KLF9 tended to round up on plastic, formed multi-layers, and exhibited dome formation [21]. Because the repertoire of genes identified in that study was limited and did not fully explain the potentially opposing functions of KLF9 on cell adhesion and proliferation, defects of which may underscore cell survival, migration, invasion, and tumorigenesis, we have now performed global expression profiling of the HEC-1-A sub-lines that over- or under-express KLF9. Results demonstrate prominent effects of KLF9 on genes encoding basement membrane and ECM proteins, cell stress response and detoxification pathway members, uterine endometrial steroid- and estrous cycle-regulated proteins, and membrane- and nuclear-associated receptors. These findings correlate with the attenuated expression of KLF9 with high endometrial tumor grade, thereby suggesting the potential involvement of KLF9 dys-regulation in both pregnancy failure and endometrial pathogenesis.

## Methods

### Cell lines

The generation and initial characterization of the cell lines used in this study were previously described [21,22]. The 4S and 9S sub-lines were derived from the original human HEC-1-A endometrial carcinoma cell line and contain a stably transfected expression plasmid encoding full-length rat KLF9, whereas the 2AS and 3AS sub-lines contain a stably transfected plasmid encoding an antisense (AS) RNA to KLF9. The primary sequence of human and rat KLF9 coding regions are 98% identical [23]. All cell lines were derived concurrently [21]. The 'sense' (S) and 'antisense' (AS) cell lines differed in relative levels (S > AS) of KLF9 protein and activity [21]. S sub-lines expressed ~2.4 fold greater immunoreactive KLF9 than AS sub-lines [21]. The 4S/2AS and 9S/3AS sub-lines were propagated separately to near confluence and RNA was harvested using TRIzol reagent (Invitrogen, Carlsbad, CA).

### Affymetrix GeneChip technology and bio-informatics

RNA was purified using the RNeasy Mini Kit (QIAGEN, Valencia, CA) followed by on-column DNA digestion with RNase-Free DNase (QIAGEN). RNA integrity was confirmed using the RNA 6000 Nano LabChip (Agilent Biotechnologies, Palo Alto, CA). Two replicate cRNA targets were generated from each HEC-1-A sub-line RNA preparation; all eight targets were made at the same time. Total cellular RNA (8 ug) was converted to cDNA using a T7-(deoxythymidine)_24 _primer and Superscript II (Life Technologies, Inc., Gaithersburg, MD). Resulting cDNA was used with the ENZO BioArray High Yield RNA Transcript labeling kit (ENZO, Farmingdale, NY) to synthesize biotin-labeled cRNA; the latter was purified on an RNeasy spin column (QIAGEN) and chemically fragmented to a size range of 35 to 200 bp. cRNAs were concurrently hybridized to HG-U133A GeneChips (Affymetrix, Santa Clara, CA). Hybridizations were performed for 16 hours, followed by incubations with streptavidin-conjugated phycoerythrin, and polyclonal anti-streptavidin antibody coupled to phycoerythrin. GeneChips were scanned using an Agilent GeneArray laser scanner and images analyzed using Affymetrix MAS 5.0 software. Bacterial sequence-derived probes on the arrays served as external controls for hybridization, whereas the housekeeping genes β-actin and GAPDH served as endogenous controls and for monitoring the quality of the RNA targets.

Unsupervised nearest-neighbor hierarchical clustering (Spotfire DecisionSite, Somerville, MA) identified a significant effect of culture condition/date of RNA collection on overall gene expression profiles. Therefore, to identify candidate KLF9 gene targets, the following was separately performed on the combinations of 4S/2AS and 9S/3AS. Intensity values of probe sets were imported into GeneSpring Gx 7.3 software for analysis. Values were processed using the Robust Multiarray Analysis algorithm for background adjustment, normalization and log_2_-transformation of perfect match values. Data were subjected to per-chip and per-gene normalization and analyzed for differences between cell lines (fold-change, S relative to AS, value of 1.3 or higher; *P *< 0.05, Student's *t *test). Transcripts that passed these filters for both the 4S/2AS and 9S/3AS cell line combinations comprised the final KLF9-regulated gene lists and were compared for gene overlaps. The final gene list (comprised only of overlapping transcripts) was annotated using NETAFFX [24] and NCBI *Entrez *[25]. The microarray data have been deposited in Gene Expression Omnibus [26] as series GSE11855.

#### Quantitative real-time RT-PCR (qRT-PCR)

One μg of total cellular RNA from each cell line was reverse-transcribed using random hexamers and MultiScribe Reverse Transcriptase in a two-step RT-PCR reaction (Applied Biosystems, Foster City, CA). Primers (Table 1) were designed using 'Primer Express' (Applied Biosystems) to yield a single amplicon; this was verified by dissociation curves. SYBR Green real-time PCR was performed with an ABI Prism 7000 Sequence Detector or Bio-Rad MyiQ Real-Time PCR Detection System. Thermal cycling conditions included pre-incubation at 50°C for 2 min, 95°C for 10 min followed by 40 PCR cycles at 95°C for 15 sec and 60°C for 1 min. Relative transcript levels were calculated using the relative standard curve method (User Bulletin #2, Applied Biosystems) and results were normalized to 18 S rRNA. Data are reported as mean ± SEM and were analyzed by one-way ANOVA (SigmaStat; Systat Software, Inc., Point Richmond, CA). *P *< 0.05 was considered to represent a significant difference.

**Table 1:** Primers used in quantitative real-time RT-PCR

Gene Name	Upstream Primer (5'-3')	Downstream Primer (5'-3')	PCR product size (bp)
18S	TCTTAGCTGAGTGTCCCGCG	ATCATGGCCTCAGTTCCG A	150
AKR7A2	AACTGGACACGGCCTTCATG	CCTTGGTGGCAATTTTCACTCTG	109
ALDH1A1	ACCCCAGGAGTCACTCAAGG	ACTGTGGGCTGGACAAAGTAG	149
BCAR3	CCTGGAAATGCCACAGATCAC	CTTCATGCAGGAGTTTGCTGAA	124
C1orf186	TAGCTTGGATAGCTCCTGCAGTTC	CATTTTTTAGTTCTCCAGGGTCAGA	101
CLIC4	TCACCAAAACACCCAGAATCAA	ACCCCTCTCCAGTGCTTCATTA	108
COL4A1	CACGGGTACTCTTTGCTCTACGT	AAGGGCATTGTGCTGAACTTG	101
COL4A2	CATGCCCTTCCTGTACTGCAA	GATGTACTTGATCTCGTCCT	133
CXCR4	CATCAGTCTGGACCGCTACC	GCAAAGATGAAGTCGGGAATAGTC	138
ER-α	CGGCATTCTACAGGCCAAATT	AGCGAGTCTCCTTGGCAGATT	
FZD5	GCTACCAGCCGTCCTTCAGT	GAAGCGTTCCATGTCGATGAG	128
KLF4	CTGCGGCAAAACCTACACAAA	GAATTTCCATCCACAGCCGT	106
KLF9	TGGCTGTGGGAAAGTCTATGG	CTCGTCTGAGCGGGAGAACT	
LAMC2	GATGGCATTCACTGCGAGAAG	TCGAGCACTAAGAGAACCTTTGG	105
LAPTM5	CATCTTTTCCATCGCCTTCATCAC	TCCACCGAGTTCATGCACTTG	102
MAPKAPK3	TCCCACCCTTCTACTCCAACA	TTCAACAGGAGGCGGATCA	141
NR3C1	AGAGGAGGAGCTACTGTGAAGG	ACTGAGCCTTTTGGAAAATCAACC	109
PAX2	CCCAGAGTGGTGTGGACAGTTT	GTAGGAAGGACGCTCAAAGACC	101
PSAT1	ACGCCTCCATGTTTCAGCAT	TGAGATTTGATGGAGCTAAGCTTCT	104
PTCH1	GTCGAGCTGTTCGGCATGAT	AGCAACGTGAACGGTGAACTC	111
RAB25	GGAGCTCTATGACCATGCTGAA	CCAGGAAGAGCAGTCCATTGTT	125
RXRA	AGGACTGCCTGATTGACAAGC	GACTCCACCTCATTCTCGTTCC	141
SLPI	GCTGTGGAAGGCTCTGGAAA	TGCCCATGCAACACTTCAAG	298

A TissueScan™ endometrial cancer qPCR array was purchased from OriGene Technologies, Inc. (Rockville, MD). This panel was comprised of a series of normalized cDNAs prepared from pathologist-verified human endometrium and endometrial tumors. Ages of tissue donors ranged from 30 to 87 years.

## Results

### Gene expression profiling

**Figure 1:**
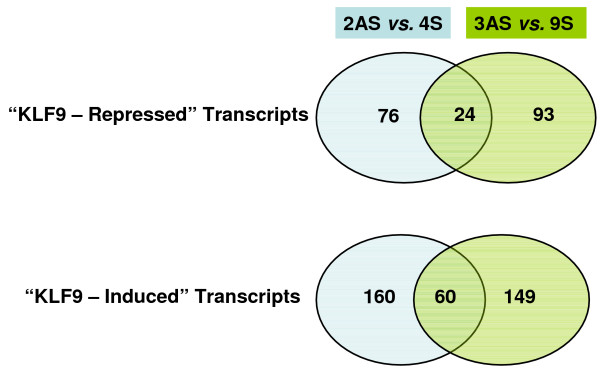
**Differentially expressed transcripts of HEC-1-A sub-lines**. Venn diagrams summarize the number of differentially expressed genes noted between 2AS and 4S sub-lines, between 3AS and 9S sub-lines, and those in common for both comparisons (final annotated gene lists are presented in additional file 1: Table 1 and additional file 2: Table 2). There were greater numbers of genes induced than repressed in concert with relative KLF9 expression.

**Figure 2:**
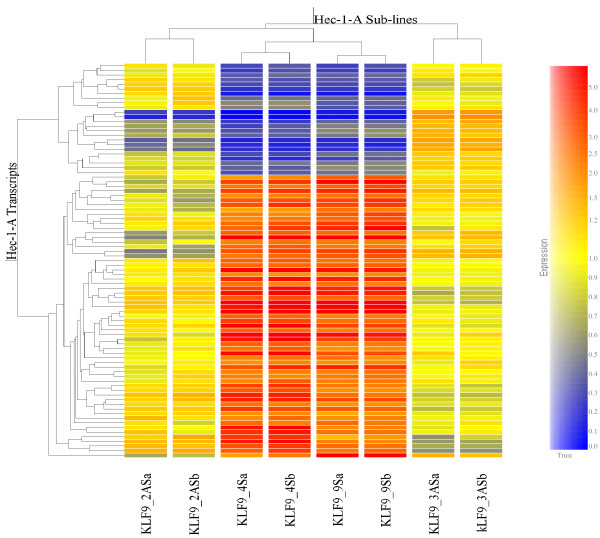
**Hierarchical clustering of differentially expressed RNAs**. The microarray data for mRNAs that were identified to be differentially expressed between S and AS sub-lines (additional file 1: Table 1 and additional file 2: Table 2) were subjected to hierarchical clustering. The transcript profiles were very similar for both S sub-lines (each run in duplicate); whereas the two AS sub-lines differed from each other and from the S sub-lines. Lower case letters signify duplicate microarrays for each sub-line.

The 4S/2AS and 9S/3AS pairs of sub-lines were grown on separate occasions to ~80% confluence. Total cellular RNAs from the four sub-lines were concurrently subjected to global gene expression profiling, with each RNA sample analyzed in duplicate. Unsupervised hierarchical clustering of gene expression profiles for all transcripts indicated a significant effect of time of experiment (i.e., when sub-lines were cultured) (data not shown). We therefore performed bio-informatic comparisons of 2AS vs. 4S and 3AS vs. 9S separately, and then searched for overlap in the differentially expressed transcripts between each paired comparison. There were more genes expressed at higher levels in KLF9 sense (S) than antisense (AS) sub-lines among the differentially expressed transcripts identified (Figure 1). However, whereas the numbers of KLF9-induced (160 and 149 for 4S and 9S, respectively) and KLF9-suppressed (76 and 93 for 2AS and 3AS, respectively) transcripts for each pair of sub-lines were comparable, less than half of these were found to overlap in both final gene lists for S (total of 60) and AS (24) sub-lines. As a conservative estimate, these overlapping transcripts were taken to reflect KLF9 activity and/or expression levels in the clonal sub-lines. Unsupervised hierarchical clustering of these signature transcripts demonstrated the close relatedness of the S sub-lines expression profiles (Figure 2). As expected, the expression profiles of the AS sub-lines differed from the S sub-lines; however, they also differed from each other (Figure 2).

Transcripts that varied in expression across the two pairs of sub-lines were annotated for their known or putative function(s), fold-change, and cellular locations (additional file 1: Table 1 and additional file 2: Table 2). KLF9 under-expression in HEC-1-A cells (i.e., AS sub-lines) caused induction in relatively few (twenty four) genes. Six of these genes/transcripts were examined by qRT-PCR and all were confirmed to be induced in the AS sub-lines (Table 2). In general, for a given transcript/gene there was excellent agreement between the fold-change calculated from microarray and that from qRT-PCR (Table 2). These KLF9-suppressed (directly or indirectly) mRNAs encode proteins which are important participants in: aldehyde metabolism (AKR7A2, ALDH1A1), regulation of the actin cytoskeleton and cell motility (e.g., ANK3, ITGB8), cellular detoxification (SULT1A1, ABCC4), cellular signaling (e.g., ACBD3, FZD5, RAB25, CALB1), and transcriptional regulation (PAX2, STAT1). In addition, two hypothetical proteins (C12orf29, C1orf186) are encoded by mRNAs whose levels were increased with KLF9 suppression.

**Table 2:** qRT-PCR confirmation of differential expression of selected transcripts^a^

	**4S *vs. *2AS (fold)**		**9S *vs. *3AS (fold)**	
**Gene Symbol**	**qRT-PCR**	**Microarray**	**qRT-PCR**	**Microarray**

	**Up-regulated**			

BCAR3	3.907	2.488	1.171	2.611
CLIC4	31.636	21.46	24.750	11.710
COL4A1	3.387	3.584	1.768	2.179
COL4A2	2.967	7.692	1.834	2.725
CXCR4	5.000	2.762	13.037	3.876
KLF4	4.532	2.110	1.816	3.185
LAMC2	1.633	3.003	2.276	3.676
LAPTM5	22.135	24.331	37.060	5.917
MAPKAPK3	3.665	3.861	7.056	3.831
NR3C1	7.111	2.841	12.500	4.386
PSAT1	1.394	2.278	2.876	6.897
PTCH1	3.572	4.310	3.008	2.242
RXRα	10.953	5.405	5.750	2.445
SLPI	26.636	9.524	4.788	11.779

	**Down-regulated**			

ALDHIAI	0.222	0.174	0.109	0.068
AKR7A2	0.647	0.491	0.264	0.387
C1orf186	0.366	0.155	0.278	0.265
FZD5	0.365	0.440	0.552	0.380
PAX2	0.145	0.247	0.041	0.382
RAB25	0.012	0.076	0.002	0.082

^a^All fold change values were statistically significant (*P *< 0.05); n = 2 replicates per cell line.

Sixty mRNAs were more abundant in 4S and 9S than 2AS and 3AS sub-lines (Figure 1). We examined a subset of these (fourteen in total) by qRT-PCR and confirmed their differential expression (Table 2). KLF9-induced mRNAs encode proteins which participate in: serine biosynthesis (e.g., PSAT1, a major progesterone-induced protein of the rabbit uterus); regulation and function of the actin cytoskeleton (COTL1, FSCN1, FXYD5, MYO10); cell adhesion, extracellular matrix and basement membrane formation (e.g., AMIGO2, COL4A1, COL4A2, LAMC2, NID2); transport (CLIC4); cellular signaling (e.g., BCAR3, MAPKAPK3); transcriptional regulation [e.g., KLF4, NR3C1 (glucocorticoid receptor), RXRα]; growth factors/cytokine actions (SLPI, BDNF); or are membrane-associated proteins and receptors (e.g., CXCR4, PTCH1); and metallothioneins (Table 2; additional file 2: Table 2). In addition, two mRNAs that encode hypothetical proteins (C10orf38, C9orf167) were increased in abundance with KLF9 over-expression.

### Novel human membrane proteins expressed in HEC-1-A cells

We confirmed the reduction in abundance of the mRNA encoding C1orf186 in the KLF9 S sub-lines by qRT-PCR (Table 2). C1orf186 protein contains 172 amino acid residues and has a predicted mol wt of 19,405 and an isoelectric point of 4.53. Its sequence bears hallmarks of a single pass trans-membrane protein with multiple potential serine, threonine and tyrosine phosphorylation sites in its intracellular face (data not shown). In humans, this transcript is highly expressed in the uterus where its abundance is regulated by stage of the menstrual cycle [25,26]. A second novel membrane protein-encoding mRNA identified from our microarray results was that for C9orf167. In contrast to C1orf186 mRNA, C9orf167 mRNA was induced by KLF9 in HEC-1-A cells. The protein encoded by C9orf167 is comprised of 422 amino acids in the human and is an uncharacterized member of the clpA/clpB family, Torsin subfamily of proteins. The paralogous protein in Chimpanzee is 99.8% similar, while the mouse protein is 84.9% similar to that for the human [25]. This transcript is ubiquitous in human and mouse tissues with abundant expression in the uterus; albeit not as dramatic as for C1orf186, uterine expression of C9orf167 also is regulated by stage of menstrual cycle in women [25,26].

### KLF9 mRNA expression in human uterine tumors

**Figure 3:**
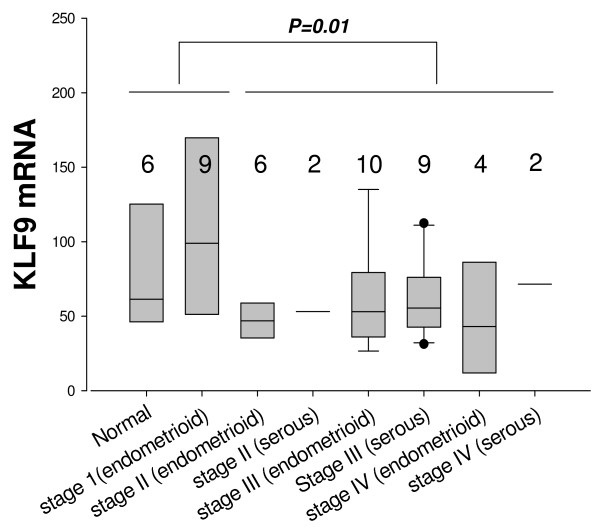
**Quantitative RT-PCR of KLF9 mRNA in human endometrium and endometrial tumors**. A normalized cDNA panel of human endometrial tumors was obtained from OriGene Technologies, Inc. This panel was comprised of cDNAs from n = 6 of normal (N) endometria, n = 9 of Stage I tumors, n = 8 of Stage II tumors, n = 19 of Stage III tumors, and n = 6 of Stage IV tumors. Shown are box plots (median, upper and lower quartiles, minimum and maximum data values) of relative abundance of KLF9 mRNA for tumors (delineated by stage and tumor type: endometrioid, serous). Sample numbers are indicated. ANOVA indicated no differences in mRNA abundance between any of the individual stages. However, a significant difference between combined stages (normal plus stage I vs. stages II, III, and IV) was noted by the Mann-Whitney Rank Sum test.

Endometrial cancer is associated with unopposed estrogen activity. In Ishikawa endometrial cancer cells, KLF9 opposed ER-α activity and decreased this protein's expression [20]. Given that KLF9 over-expression increased proliferation [21], and induced the expression of transcripts encoding multiple ECM and basement membrane components for increased adhesion to substratum (additional file 1: Table 1 and additional file 2: Table 2) in HEC-1-A cells, it was of interest to examine human endometrial tumors for expression of KLF9 mRNA. A reduction in KLF9 mRNA abundance in combined stages II through IV tumors, compared to normal endometrium and stage I tumors was observed (Fig. 3).

## Discussion

The present study extended our initial characterization of HEC-1-A sub-lines which were genetically engineered to have enhanced or reduced expression of KLF9, relative to the parental HEC-1-A cell line [21]. Concordant with KLF9's dual function as a transcriptional activator and transcriptional repressor, a range of genes involved in distinct signal transduction pathways was induced or repressed by KLF9. Using the unbiased microarray approach, we found significantly more KLF9-induced than KLF9-repressed transcripts in HEC-1-A sub-lines. Although the biological significance of this finding is unclear, it raises the important question of whether cellular context defines in part, the transcriptional direction of KLF9 activity, and whether this difference may be underscored by distinct interactions of KLF9 with various co-activators and co-repressors also present. The degree of overlap of transcripts noted between the two pairs of compared sub-lines was less than anticipated and may reflect the arbitrary cut-offs used for bio-informatic analysis, the effects of small differences in culture conditions on mRNA expression, and the non-specific effects of the process of clonal sub-line derivation.

In previous studies, we showed that the S lines with higher KLF9 expression and mitotic index grew as monolayers, whereas the AS lines with lower KLF9 expression and mitotic index tended to round up on plastic, formed multi-layers, and exhibited dome formation [21], the latter potentially indicative of enhanced tumor invasive capability and metastasis. Our current findings identified putative KLF9-regulated genes whose proteins are known to contribute to basement membrane formation, ECM formation, cell adhesion, and cytoskeletal organization and regulation. Of note was the KLF9 up-regulated expression of the LAMC2 gene, encoding the basement membrane laminin gamma 2 protein, which is important for epithelial anchoring to substratum. While regulation of other laminin genes by other KLF family members has been previously reported [27,28], our findings constitute the first linkage of KLF9 and laminin gene expression for any cell type. Basement membranes and extracellular matrix are dynamic entities that do more than just passively anchor cells to surfaces; they are, for example, important modifiers of cellular growth, differentiation, apoptosis, *anoikis *and migration. Thus, loss of KLF9 which regulates secreted and membrane-associated proteins such as laminin, might constitute an early event in the epithelial-mesenchymal transition, leading to invasion and subsequent metastasis. Consistent with this, endometrial tumors of higher grades (and with greater invasion and metastasis) had decreased KLF9 gene expression compared to normal endometrium and stage I tumors. While this association is not particularly strong and requires additional confirmation with increased sample size and evaluation at the protein level, our data mirrors a recent report that decreased KLF9 expression also is a feature of increased grade of colon tumors [29].

Previously, we observed increased KLF5 mRNA abundance in HEC-1-A cells after KLF9 over-expression, with no differences in mRNA level for Sp1, the best characterized transcriptional activator of the Sp/KLF family [22]. In the present study, this positive association with KLF9 was similarly noted for KLF4. In colon crypt epithelium, KLF4 mediates growth arrest and induction of the colonic epithelial phenotype, whereas KLF5 stimulates proliferation of crypt progenitor cells [30,31]. By contrast, KLF13, the most closely related family member to KLF9 [1] was found to be inversely associated with KLF9 expression in uteri of KLF9 null mice, possibly reflective of partial functional compensation [15]. While the roles of KLF4, KLF5 and KLF 13 in normal and tumorigenic uterine epithelium and their regulation by KLF9 are as yet unknown, the presence of all three members in the uterus suggests a KLF network where these proteins' cooperative, opposing, or compensatory functions in the physiological contexts of uterine remodeling, embryo implantation, tumorigenesis, and stem cell regulation may be manifest. Indeed, functional KLF networks have been reported in the maintenance of the intestinal epithelial cell phenotype [32], self renewal of embryonic stem cells [4], and secondary antibody response in memory B cells [14].

Previous work from our laboratories has implicated KLF9 in progesterone and estrogen actions in the uterus [15,17,19,33]. The present results further support these linkages. PSAT1, an *in vivo *progesterone-induced gene in rabbit uterus [34], was more highly expressed in the KLF9 over-expressing HEC-1-A cells. We also found repression of ALDH1 gene expression and increased levels of CXCR4 and SLPI gene transcripts in KLF9 S cells. In ovariectomized mice, uterine ALDH1A1 mRNA was rapidly induced by progesterone, whereas CXCR4 transcripts were down-regulated by chronic progesterone treatment [35]. SLPI is a progesterone-induced, secreted anti-protease with anti-microbial and mitogenic activities for uterine epithelial cells [36-38]. Expression of the uterine SLPI gene/protein is increased around the time of implantation in rodents, pigs, primates and humans in response to estrogen and progesterone [39-43]. Given the distinct patterns of expression of CXCR4, ALDH1A1 and SLPI genes with KLF9 in vitro and with progesterone in vivo, results suggest the functional contribution of KLF9 to progesterone receptor signaling. In this regard, a recent study from our laboratories demonstrated the progesterone-dependent co-recruitment of KLF9 and the progesterone receptor to the SLPI promoter, concomitant with induction of this gene's expression, in Ishikawa endometrial cancer cells [19].

We observed that Patched 1 (PTCH1) transcripts were induced in KLF9 S sub-lines. This gene encodes the receptor for the morphogen Sonic Hedgehog (SHH), a known stimulator of endometrial cell proliferation *in vitro *[44]. The related molecule, Indian Hedgehog (IHH), is a progesterone-induced molecule in the mouse uterus and mediates effects of progesterone on uterine epithelial-stromal cell interactions essential for implantation [45]. CXCR4 encodes a chemokine receptor that is up-regulated during the window of implantation in human endometrium and which may facilitate blastocyst adhesion to the uterine surface [46-49]. The CXCR4 ligand, namely stromal cell-derived factor-1α (SDF-1α), promotes proliferation of HEC-1-A cells *in vitro *through Akt and ERK1/2 signaling pathways [50]. Thus, up-regulated expression of PTCH1 and CXCR4 mRNAs with over-expression of KLF9 may explain the increased mitogenesis of KLF9 S cell lines observed *in vitro *[21,22]. CXCR4 is also a co-receptor for HIV-1, and uterine expression of HIV receptors/co-receptors may underlie HIV transmission to women [47]. Moreover, KLF9 is itself a transcriptional inducer of the HIV-1 LTR [7]. The latter data raise the provocative question of whether KLF9 affects uterine and vaginal uptake and replication of HIV-1 *in vivo*, and if so, whether KLF9 might constitute a genetic modifier of HIV risk. Further studies are required to evaluate this possibility as well as the mode of regulation of CXCR4 by KLF9 in the female reproductive tract.

The power of microarrays is that they often reveal new participants and novel functional connections in specific physiological contexts. We found several interesting examples of these in the present work. Brain-derived neurotrophic factor (BNDF) was observed to be up-regulated in KLF9 S sub-lines, suggesting BNDF as a downstream effector of KLF9 signaling. Recently, this soluble factor was implicated as a mediator of cyclic changes in sympathetic innervation in the uterus and in uterine-embryo communication [51,52]. We also found that the Frizzled gene FZD5, which encodes the receptor for the secreted glycoprotein Wnt 5B, normally expressed in human endometrium [53] is suppressed by KLF9 in HEC-1-A cells; suggesting the contribution of KLF9 to the control of this growth factor-receptor pathway.

We noted the induction of several metallothionein genes (MT1H, MTH1X, and MT2A) with KLF9 over-expression. Metallothioneins are expressed in cyclic fashion in human endometrium with most abundant expression during the secretory phase, where they may play protective roles during cell stress [53,54]. The hyaluronic acid receptor CD44 mRNA also was increased in abundance with KLF9 over-expression. This molecule is regulated in human endometrium by stage of menstrual cycle (maximal during mid and late secretory phases) [55]. Versican mRNAs are up-regulated in human endometrium during the mid-secretory phase [53]; accordingly, we noted increased expression of this gene with KLF9 over-expression. Thus, with these genes, KLF9 transactivation was correlated with secretory phase uterine expression. However, the apparent repression of SULT1A1 gene by KLF9 in HEC-1-A cells contrasted with the increased abundance of this gene during the secretory relative to the proliferative phase of the human endometrium [56].

A prominent role for KLF9 in cellular response to chemotherapeutic agents and other cell stressors is suggested from the present results. As noted above, metallothioneins, which were KLF9-induced, likely have a protective role during endometrial cell stress [54]. KLF9 over-expressing HEC-1-A cells had increased mRNA expression of BCAR3 and PSAT1, proteins previously implicated in the acquisition of tamoxifen resistance by breast cancer cells [57,58]. PSAT1 also is involved in the process by which colon cancer cells acquire chemotherapy resistance [59]. Expression of the KLF9 gene target SLPI is induced in endometria of women receiving tamoxifen [60]. Thus, KLF9 through several of its downstream targets may participate in the pathway(s) and network(s) by which tamoxifen becomes estrogenic and thereby, tumorigenic for a subset of pre-disposed uterine endometrial epithelial cells.

KLF9 over-expressing HEC-1-A cells had increased abundance of MAPKAPK3, TRIB3 and ELK3 mRNAs; the corresponding proteins participate in cell stress-response pathways [61]. Interestingly, MAPKAPK3, a Ser-Thr kinase, is activated by serum and other growth inducers and is a point of convergence of ERK, p38MAP kinase and Jun N-terminal kinase (JNK) signaling pathways [61]. By inducing expression of MAPKAPK3, KLF9 may enhance basal and/or overall activity of one or more of these important pathways. Consistent with these findings, we previously demonstrated that KLF9 over-expression caused: 1) HEC-1-A cells to become more mitogenically responsive to serum and TGF-β1 [21], and 2) the IGFBP2 gene and its promoter to be potently induced by serum [22]. Moreover, curcumin, an inhibitor of JNK, blocked the inductive effect of KLF9 on serum-stimulation of the IGFBP2 gene promoter [22]. Another group has reported that acetaldehyde induced KLF9 expression in rat hepatic stellate cells via activation of the JNK pathway [62]. Altogether, these observations invoke KLF9 as a nuclear participant in the JNK pathway and the cellular responses that it subserves. Thus, in keeping with a functional KLF network in endometrial epithelial cells, KLF9 joins its nuclear brethren, KLF4 and KLF5, as potentially important mediators of cellular stress response [2].

Results implicate KLF9 in the transcriptional regulation of key pathways that sub-serve proliferation, adhesion, migration, stress, response to chemotherapeutic agents, embryo attachment and implantation, and uterine remodeling. Coupled with the ability of KLF9 to interact with other members of the Sp/KLF family, progesterone receptors, and receptor co-activators and co-repressors, our data suggest expanded possibilities for KLF9 to affect distinct physiological processes as well as the genesis of endometrial tumors. Given that KLF9 null mice are viable with a relatively mild uterine phenotype, further study of KLF9 is warranted to clearly define its unique functions in normal uterine biology and in endometrial neoplasia distinct from those potentially compensated by closely related KLFs.

## Conclusion

Microarray profiling of HEC-1-A sub-lines, differing in relative expression of KLF9, identified a network of genes downstream of this transcription factor. The nature of the participants in this network implicates KLF9 in control of endometrial cell adhesion to substratum, tumor cell migration and invasion, cell stress responses, embryo attachment to endometrium, and uterine remodeling. Additional studies of the complex, multi-factorial role of KLF9 in the uterus are therefore warranted.

## Competing interests

The authors declare that they have no competing interests.

## Authors' contributions

FAS and RCMS conceived and designed the study. YS performed bioinformatics, gene annotation, and qRT-PCR data analyses. RX conducted the microarray experiment. ZZ performed the human endometrial tumor gene expression study. FAS prepared the initial draft of the manuscript. All co-authors provided inputs during final manuscript preparation.

## Supplementary Material

Additional file 1 Table 1: RNAs that are more highly expressed in KLF9 AS HEC-1-A sub-lines. Click here for file

Additional file 2 Table 2: RNAs that are more highly expressed in KLF9 S HEC-1-A sub-lines. Click here for file

## References

[B1] Suske G, Bruford E, Philipsen S (2005). Mammalian SP/KLF transcription factors: bring in the family. Genomics.

[B2] McConnell BB, Ghaleb AM, Nandan MO, Yang VW (2007). The diverse functions of Krüppel-like factors 4 and 5 in epithelial biology and pathobiology. Bioessays.

[B3] Takahashi K, Tanabe K, Ohnuki M, Narita M, Ichisaka T, Tomoda K, Yamanaka S (2007). Induction of pluripotent stem cells from adult human fibroblasts by defined factors. Cell.

[B4] Jiang J, Chan YS, Loh YH, Cai J, Tong GQ, Lim CA, Robson P, Zhong S, Ng HH (2008). A core Klf circuitry regulates self-renewal of embryonic stem cells. Nat Cell Biol.

[B5] Park IH, Zhao R, West JA, Yabuuchi A, Huo H, Ince TA, Lerou PH, Lensch MW, Daley GQ (2008). Reprogramming of human somatic cells to pluripotency with defined factors. Nature.

[B6] Imataka H, Sogawa K, Yasumoto K, Kikuchi Y, Sasano K, Kobayashi A, Hayami M, Fujii-Kuriyama Y (1992). Two regulatory proteins that bind to the basic transcription element (BTE), a GC box sequence in the promoter region of the rat P-4501A1 gene. EMBO J.

[B7] Imataka H, Mizuno A, Fujii-Kuriyama Y, Hayami M (1993). Activation of the human immunodeficiency virus type 1 long terminal repeat by BTEB, a GC box-binding transcription factor. AIDS Res Hum Retroviruses.

[B8] Foti D, Stroup D, Chiang JY (1998). Basic transcription element binding protein (BTEB) transactivates the cholesterol 7 alpha-hydroxylase gene (CYP7A). Biochem Biophys Res Commun.

[B9] Denver RJ, Ouellet L, Furling D, Kobayashi A, Fujii-Kuriyama Y, Puymirat J (1999). Basic transcription element-binding protein (BTEB) is a thyroid hormone-regulated gene in the developing central nervous system. Evidence for a role in neurite outgrowth. J Biol Chem.

[B10] Martel J, Cayrou C, Puymirat J (2002). Identification of new thyroid hormone-regulated genes in rat brain neuronal cultures. Neuroreport.

[B11] Cayrou C, Denver RJ, Puymirat J (2002). Suppression of the basic transcription element-binding protein in brain neuronal cultures inhibits thyroid hormone-induced neurite branching. Endocrinology.

[B12] Morita M, Kobayashi A, Yamashita T, Shimanuki T, Nakajima O, Takahashi S, Ikegami S, Inokuchi K, Yamashita K, Yamamoto M, Fujii-Kuriyama Y (2003). Functional analysis of basic transcription element binding protein by gene targeting technology. Mol Cell Biol.

[B13] Simmen FA, Xiao R, Velarde MC, Nicholson RD, Bowman MT, Fujii-Kuriyama Y, Oh SP, Simmen RCM (2007). Dysregulation of intestinal crypt cell proliferation and villus cell migration in mice lacking Kruppel-like factor 9. Am J Physiol Gastrointest Liver Physiol.

[B14] Good KL, Tangye SG (2007). Decreased expression of Kruppel-like factors in memory B cells induces the rapid response typical of secondary antibody responses. Proc Natl Acad Sci USA.

[B15] Simmen RCM, Eason RR, McQuown JR, Linz AL, Kang TJ, Chatman L, Till SR, Fujii-Kuriyama Y, Simmen FA, Oh SP (2004). Subfertility, uterine hypoplasia, and partial progesterone resistance in mice lacking the Kruppel-like factor 9/basic transcription element-binding protein-1 (Bteb1) gene. J Biol Chem.

[B16] Zeng Z, Velarde MC, Simmen FA, Simmen RCM (2008). Delayed parturition and altered myometrial progesterone receptor isoform a expression in mice null for Krüppel-like factor 9. Biol Reprod.

[B17] Velarde MC, Geng Y, Eason RR, Simmen FA, Simmen RCM (2005). Null mutation of Kruppel-like factor9/basic transcription element binding protein-1 alters peri-implantation uterine development in mice. Biol Reprod.

[B18] Zhang XL, Zhang D, Michel FJ, Blum JL, Simmen FA, Simmen RCM (2003). Selective interactions of Kruppel-like factor 9/basic transcription element-binding protein with progesterone receptor isoforms A and B determine transcriptional activity of progesterone-responsive genes in endometrial epithelial cells. J Biol Chem.

[B19] Velarde MC, Iruthayanathan M, Eason RR, Zhang D, Simmen FA, Simmen RCM (2006). Progesterone receptor transactivation of the secretory leukocyte protease inhibitor gene in Ishikawa endometrial epithelial cells involves recruitment of Krüppel-like factor 9/basic transcription element binding protein-1. Endocrinology.

[B20] Velarde MC, Zeng Z, McQuown JR, Simmen FA, Simmen RCM (2007). Kruppel-like factor 9 is a negative regulator of ligand-dependent estrogen receptor (alpha) signaling in Ishikawa endometrial adenocarcinoma cells. Mol Endocrinol.

[B21] Zhang XL, Simmen FA, Michel FJ, Simmen RCM (2001). Increased expression of the Zn-finger transcription factor BTEB1 in human endometrial cells is correlated with distinct cell phenotype, gene expression patterns, and proliferative responsiveness to serum and TGF-beta1. Mol Cell Endocrinol.

[B22] Simmen RCM, Zhang XL, Michel FJ, Min SH, Zhao G, Simmen FA (2002). Molecular markers of endometrial epithelial cell mitogenesis mediated by the Sp/Kruppel-like factor BTEB1. DNA Cell Biol.

[B23] Ohe N, Yamasaki Y, Sogawa K, Inazawa J, Ariyama T, Oshimura M, Fujii-Kuriyama Y (1993). Chromosomal localization and cDNA sequence of human BTEB, a GC box binding protein. Somat Cell Mol Genet.

[B24] The NetAffx Analysis Center. http://www.affymetrix.com/analysis/index.affx.

[B25] *Entrez *The Life Sciences Search Engine. http://www.ncbi.nlm.nih.gov/sites/gquery.

[B26] Gene Expression Omnibus. http://www.ncbi.nlm.nih.gov/geo/.

[B27] Higaki Y, Schullery D, Kawata Y, Shnyreva M, Abrass C, Bomsztyk K (2002). Synergistic activation of the rat laminin gamma1 chain promoter by the gut-enriched Kruppel-like factor (GKLF/KLF4) and Sp1. Nucleic Acids Res.

[B28] Piccinni SA, Bolcato-Bellemin AL, Klein A, Yang VW, Kedinger M, Simon-Assmann P, Lefebvre O (2004). Kruppel-like factors regulate the Lama1 gene encoding the laminin alpha1 chain. J Biol Chem.

[B29] Kang L, Lü B, Xu J, Hu H, Lai M (2008). Downregulation of Krüppel-like factor 9 in human colorectal cancer. Pathol Int.

[B30] Shields JM, Christy RJ, Yang VW (1996). Identification and characterization of a gene encoding a gut-enriched Krüppel-like factor expressed during growth arrest. J Biol Chem.

[B31] Shie JL, Chen ZY, O'Brien MJ, Pestell RG, Lee ME, Tseng CC (2000). Role of gut-enriched Krüppel-like factor in colonic cell growth and differentiation. Am J Physiol Gastrointest Liver Physiol.

[B32] Dang DT, Zhao W, Mahatan CS, Geiman DE, Yang VW (2002). Opposing effects of Krüppel-like factor 4 (gut-enriched Krüppel-like factor) and Krüppel-like factor 5 (intestinal-enriched Krüppel-like factor) on the promoter of the Krüppel-like factor 4 gene. Nucleic Acids Res.

[B33] Zhang D, Zhang XL, Michel FJ, Blum JL, Simmen FA, Simmen RCM (2002). Direct interaction of the Krüppel-like family (KLF) member, BTEB1, and PR mediates progesterone-responsive gene expression in endometrial epithelial cells. Endocrinology.

[B34] Misrahi M, Atger M, Milgrom E (1987). A novel progesterone-induced messenger RNA in rabbit and human endometria. Cloning and sequence analysis of the complementary DNA. Biochemistry.

[B35] Jeong JW, Lee KY, Kwak I, White LD, Hilsenbeck SG, Lydon JP, DeMayo FJ (2005). Identification of murine uterine genes regulated in a ligand-dependent manner by the progesterone receptor. Endocrinology.

[B36] Badinga L, Michel FJ, Simmen RCM (1999). Uterine-associated serine protease inhibitors stimulate deoxyribonucleic acid synthesis in porcine endometrial glandular epithelial cells of pregnancy. Biol Reprod.

[B37] Zhang D, Simmen RC, Michel FJ, Zhao G, Vale-Cruz D, Simmen FA (2002). Secretory leukocyte protease inhibitor mediates proliferation of human endometrial epithelial cells by positive and negative regulation of growth-associated genes. J Biol Chem.

[B38] Velarde MC, Parisek SI, Eason RR, Simmen FA, Simmen RCM (2005). The secretory leukocyte protease inhibitor gene is a target of epidermal growth factor receptor action in endometrial epithelial cells. J Endocrinol.

[B39] Reed KL, Blaeser LL, Dantzer V, Green ML, Simmen RCM (1998). Control of secretory leukocyte protease inhibitor gene expression in the porcine periimplantation endometrium: a case of maternal-embryo communication. Biol Reprod.

[B40] King AE, Morgan K, Sallenave JM, Kelly RW (2003). Differential regulation of secretory leukocyte protease inhibitor and elafin by progesterone. Biochem Biophys Res Commun.

[B41] Okulicz WC, Ace CI (2003). Temporal regulation of gene expression during the expected window of receptivity in the rhesus monkey endometrium. Biol Reprod.

[B42] Ace CI, Okulicz WC (2004). Microarray profiling of progesterone-regulated endometrial genes during the rhesus monkey secretory phase. Reprod Biol Endocrinol.

[B43] Chen D, Xu X, Cheon YP, Bagchi MK, Bagchi IC (2004). Estrogen induces expression of secretory leukocyte protease inhibitor in rat uterus. Biol Reprod.

[B44] Feng YZ, Shiozawa T, Miyamoto T, Kashima H, Kurai M, Suzuki A, Ying-Song J, Konishi I (2007). Overexpression of hedgehog signaling molecules and its involvement in the proliferation of endometrial carcinoma cells. Clin Cancer Res.

[B45] Lee K, Jeong J, Kwak I, Yu CT, Lanske B, Soegiarto DW, Toftgard R, Tsai MJ, Tsai S, Lydon JP, DeMayo FJ (2006). Indian hedgehog is a major mediator of progesterone signaling in the mouse uterus. Nat Genet.

[B46] Dominguez F, Galan A, Martin JJ, Remohi J, Pellicer A, Simón C (2003). Hormonal and embryonic regulation of chemokine receptors CXCR1, CXCR4, CCR5 and CCR2B in the human endometrium and the human blastocyst. Mol Hum Reprod.

[B47] Yeaman GR, Howell AL, Weldon S, Demian DJ, Collins JE, O'Connell DM, Asin SN, Wira CR, Fanger MW (2003). Human immunodeficiency virus receptor and coreceptor expression on human uterine epithelial cells: regulation of expression during the menstrual cycle and implications for human immunodeficiency virus infection. Immunology.

[B48] Hess AP, Hamilton AE, Talbi S, Dosiou C, Nyegaard M, Nayak N, Genbecev-Krtolica O, Mavrogianis P, Ferrer K, Kruessel J, Fazleabas AT, Fisher SJ, Giudice LC (2007). Decidual stromal cell response to paracrine signals from the trophoblast: amplification of immune and angiogenic modulators. Biol Reprod.

[B49] Tapia A, Gangi LM, Zegers-Hochschild F, Balmaceda J, Pommer R, Trejo L, Pacheco IM, Salvatierra AM, Henríquez S, Quezada M, Vargas M, Ríos M, Munroe DJ, Croxatto HB, Velasquez L (2008). Differences in the endometrial transcript profile during the receptive period between women who were refractory to implantation and those who achieved pregnancy. Hum Reprod.

[B50] Zhao D, Li XP, Gao M, Zhao C, Wang JL, Wei LH (2006). Stromal cell-derived factor 1alpha stimulates human endometrial carcinoma cell growth through the activation of both extracellular signal-regulated kinase 1/2 and Akt. Gynecol Oncol.

[B51] Krizsan-Agbas D, Pedchenko T, Hasan W, Smith PG (2003). Oestrogen regulates sympathetic neurite outgrowth by modulating brain derived neurotrophic factor synthesis and release by the rodent uterus. Eur J Neurosci.

[B52] Kawamura K, Kawamura N, Fukuda J, Kumagai J, Hsueh AJ, Tanaka T (2007). Regulation of preimplantation embryo development by brain-derived neurotrophic factor. Dev Biol.

[B53] Talbi S, Hamilton AE, Vo KC, Tulac S, Overgaard MT, Dosiou C, Le Shay N, Nezhat CN, Kempson R, Lessey BA, Nayak NR, Giudice LC (2006). Molecular phenotyping of human endometrium distinguishes menstrual cycle phases and underlying biological processes in normo-ovulatory women. Endocrinology.

[B54] Ioachim EE, Kitsiou E, Carassavoglou C, Stefanaki S, Agnantis NJ (2000). Immunohistochemical localization of metallothionein in endometrial lesions. J Pathol.

[B55] Yaegashi N, Fujita N, Yajima A, Nakamura M (1995). Menstrual cycle dependent expression of CD44 in normal human endometrium. Hum Pathol.

[B56] Rubin GL, Harrold AJ, Mills JA, Falany CN, Coughtrie MW (1999). Regulation of sulphotransferase expression in the endometrium during the menstrual cycle, by oral contraceptives and during early pregnancy. Mol Hum Reprod.

[B57] Schrecengost RS, Riggins RB, Thomas KS, Guerrero MS, Bouton AH (2007). Breast cancer antiestrogen resistance-3 expression regulates breast cancer cell migration through promotion of p130Cas membrane localization and membrane ruffling. Cancer Res.

[B58] Martens JW, Nimmrich I, Koenig T, Look MP, Harbeck N, Model F, Kluth A, Bolt-de Vries J, Sieuwerts AM, Portengen H, Meijer-Van Gelder ME, Piepenbrock C, Olek A, Höfler H, Kiechle M, Klijn JG, Schmitt M, Maier S, Foekens JA (2005). Association of DNA methylation of phosphoserine aminotransferase with response to endocrine therapy in patients with recurrent breast cancer. Cancer Res.

[B59] Vie N, Copois V, Mollevi C, Denis V, Bec N, Robert B, Fraslon C, Conseiller E, Molina F, Larroque C, Martineau P, Del Rio M, Gongora C (2008). Overexpression of phosphoserine aminotransferase PSAT1 stimulates cell growth and increases chemoresistance of colon cancer cells. Mol Cancer.

[B60] Gielen SC, Kühne LC, Ewing PC, Blok LJ, Burger CW (2005). Tamoxifen treatment for breast cancer enforces a distinct gene-expression profile on the human endometrium: an exploratory study. Endocr Relat Cancer.

[B61] Ludwig S, Engel K, Hoffmeyer A, Sithanandam G, Neufeld B, Palm D, Gaestel M, Rapp UR (1996). 3pK, a novel mitogen-activated protein (MAP) kinase-activated protein kinase, is targeted by three MAP kinase pathways. Mol Cell Biol.

[B62] Chen A, Davis BH (2000). The DNA binding protein BTEB mediates acetaldehyde-induced, jun N-terminal kinase-dependent alphaI(I) collagen gene expression in rat hepatic stellate cells. Mol Cell Biol.

