# Supplementary material for: The Krüppel-like factor 9 (KLF9) network in HEC-1-A endometrial carcinoma cells suggests the carcinogenic potential of dys-regulated KLF9 expression
Source: Reprod Biol Endocrinol. 2008 Sep 10;6:41. doi: 10.1186/1477-7827-6-41 (PMC2542371; doi:10.1186/1477-7827-6-41)
Supplement: Additional file 1 — Table 1: RNAs that are more highly expressed in KLF9 AS HEC-1-A sub-lines. [file 1477-7827-6-41-S1.pdf]

Table 1. RNAs that are more highly expressed in KLF9 AS HEC-1-A sub-lines.

Gene Symbol	Gene Name	Affy ID	9S vs. 3AS (fold)	4S vs. 2AS (fold)	Category/Function/Location
			**Metabolism**		
AKR7A2	Aldo-keto reductase family 7, member A2 (aflatoxin aldehyde reductase)	214259_S_AT	0.387	0.491	Aldehyde metabolism, detoxification of aldehydes and ketones
ALDH1A1	Aldehyde dehydrogenase 1 family, member A1	212224_AT	0.068	0.174	Aldehyde and alcohol metabolism
GLS	Glutaminase	203158_S_AT	0.135	0.349	D-glutamine and D-glutamate metabolism
IDS	Iduronate 2-sulfatase (Hunter syndrome)	206342_X_AT	0.334	0.407	Heparan sulfate and dermatan sulfate metabolism
MLSTD1	Male sterility domain containing 1	220615_S_AT	0.463	0.348	Fatty alcohol biosynthesis
OGDHL	Oxoglutarate dehydrogenase-like	219277_S_AT	0.282	0.430	Succinyl-transferring
SULT1A1	Sulfotransferase family, cytosolic, 1A, phenol-preferring, member 1	215299_X_AT	0.394	0.499	Sulfur metabolism, phase II metabolism of drugs and xenobiotics
			**Cytoskeleton, motility, adhesion**		
ANK3	Ankyrin 3, node of Ranvier (ankyrin G)	206385_S_AT	0.334	0.325	Cytoskeleton-membrane protein interactions
CD24	CD24 molecule	208650_S_AT	0.033	0.148	Membrane-associated sialoglycoprotein
CYFIP2	Cytoplasmic FMR1 interacting protein 2	215785_S_AT	0.466	0.452	Cytoskeleton regulation, apoptosis
GNE	Glucosamine (UDP-N-acetyl)-2-epimerase/N-acetylmannosamine kinase	205042_AT	0.100	0.295	Sialic acid biosynthesis, cell adhesion, signaling
ITGB8	Integrin, beta 8	205816_AT	0.383	0.486	Cell adhesion, migration, and matrix association
MFAP3L	Microfibrillar-associated protein 3-like	205442_AT	0.368	0.348	Integral to membrane
			**Transport**		
ABCC4	ATP-binding cassette, sub-family C (CFTR/MRP), member 4	203196_AT	0.290	0.308	Transporter, cellular detoxification
ACBD3	Acyl-Coenzyme A binding domain containing 3	202324_S_AT	0.233	0.299	Golgi resident protein, hormonal regulation of steroidogenesis, numb signaling
SEC14L1	SEC14-like 1 (S. cerevisiae)	202083_S_AT	0.457	0.500	Intracellular transport
			**Signal transduction**		
FZD5	Frizzled homolog 5 (Drosophila)	221245_S_AT	0.380	0.440	Wnt5A receptor
RAB25	RAB25, member RAS oncogene family	218186_AT	0.082	0.076	Transcytosis, migration, invasion, anti-apoptosis

		Transcription factors			
PAX2	Paired box 2	206228_AT	0.382	0.247	
STAT1	Signal transducer and activator of transcription 1, 91kDa	209969_S_AT	0.322	0.495	
		Other			
CALB1	Calbindin 1, 28kDa	205625_S_AT	0.092	0.428	Calcium binding
C12orf29	Chromosome 12 open reading frame 29	213701_AT	0.406	0.343	
C1orf186	Chromosome 1 open reading frame 186	222281_S_AT	0.265	0.155	Putative membrane-associated
METTL7A	Methyltransferase like 7A	207761_S_AT	0.266	0.211	
