# Supplementary material for: The Krüppel-like factor 9 (KLF9) network in HEC-1-A endometrial carcinoma cells suggests the carcinogenic potential of dys-regulated KLF9 expression
Source: Reprod Biol Endocrinol. 2008 Sep 10;6:41. doi: 10.1186/1477-7827-6-41 (PMC2542371; doi:10.1186/1477-7827-6-41)
Supplement: Additional file 2 — Table 2: RNAs that are more highly expressed in KLF9 S HEC-1-A sub-lines. [file 1477-7827-6-41-S2.pdf]

Table 2. RNAs that are more highly expressed in KLF9 S HEC-1-A sub-lines.

Gene Symbol	Gene Name	Affy ID	9S vs. 3AS (fold)	4S vs. 2AS (fold)	Category/Function/Location
	Metabolism				
NT5E	5'-nucleotidase, ecto (CD73)	203939_AT	2.985	6.410	Generation of adenosine
PSAT1	Phosphoserine aminotransferase 1	220892_S_AT	6.897	2.278	Serine biosynthesis, “progesterone-induced protein”
UPP1	Uridine phosphorylase 1	203234_AT	3.257	2.786	Pyrimidine metabolism
	Cytoskeleton, motility, adhesion				
AMIGO2	Adhesion molecule with Ig-like domain 2	222108_AT	2.740	2.227	Cell adhesion and migration
CD44	CD44 antigen (Indian blood group system)	204489_S_AT	3.145	2.101	Cell adhesion and migration, signal transduction, hyaluronic acid receptor
COL4A1	Collagen, type IV, alpha 1	211980_AT	2.179	3.584	Basement membrane
COL4A2	Collagen, type IV, alpha 2	211964_AT	2.725	7.692	Basement membrane
COTL1	Coactosin-like 1 (Dictyostelium)	221059_S_AT	3.663	2.770	Regulation of actin cytoskeleton
FSCN1	Fascin homolog 1, actin-bundling protein (Strongylocentrotus purpuratus)	201564_S_AT	2.618	2.950	Actin cytoskeleton, proliferation
FXYD5	FXYD domain containing ion transport regulator 5	218084_X_AT	3.356	3.484	Actin cytoskeleton, cell motility
HSPB1	Heat shock 27kDa protein 1	201841_S_AT	2.674	5.587	Anti-apoptosis, cell motility
LAMC2	Laminin, gamma 2	202267_AT	3.676	3.003	Basement membrane, cell adhesion
MYO10	Myosin X	201976_S_AT	2.421	9.174	Cytoskeleton, signal transduction
NID2	Nidogen 2 (osteonidogen)	204114_AT	3.413	6.173	Basement membrane
PCDH1	Protocadherin 1 (cadherin-like 1)	203918_AT	2.506	2.688	Cell adhesion
TM4SF1	Transmembrane 4 L six family member 1	209386_AT	3.125	4.115	Signal transduction, cell growth and motility (tetraspanin family)
TNC	Tenascin C (hexabrachion)	201645_AT	5.291	18.904	Cell adhesion
VCAN	Versican	215646_S_AT	6.494	6.211	Cell adhesion, extracellular matrix
	Transport				
ATP2C2	ATPase, Ca^{++} transporting, type 2C, member 2	206043_S_AT	2.257	2.732	Calcium transport, secretory pathway
ATP9A	ATPase, Class II, type 9A	212062_AT	2.024	4.975	Phospholipid transport

CLIC4	Chloride intracellular channel 4	201560_AT	11.710	21.459	Chloride transport, pro-apoptosis
KCNN4	Potassium intermediate/small conductance calcium-activated channel, subfamily N, member 4	204401_AT	2.538	3.610	Potassium channel
NAPA	N-ethylmaleimide-sensitive factor attachment protein, alpha	208751_AT	6.993	2.132	Intracellular protein transport
SLC25A37	Solute carrier family 25, member 37	221920_S_AT	2.299	2.994	Iron transport, mitochondrial
SLC26A2	Solute carrier family 26 (sulfate transporter), member 2	205097_AT	3.623	3.846	Sulfate transporter
	**Signal transduction**				
BCAR3	Breast cancer anti-estrogen resistance 3	204032_AT	2.611	2.488	GDP exchange factor, stress pathway, motility
MAPKAPK3	Mitogen-activated protein kinase-activated protein kinase 3	202787_S_AT	3.831	3.861	Serine-threonine kinase, mitogenic and stress pathways
PSD3	Pleckstrin and Sec7 domain containing 3	218613_AT	4.082	3.125	
ROR1	Receptor tyrosine kinase-like orphan receptor 1	205805_S_AT	4.630	3.049	ROR subfamily of receptors
SH2B3	SH2B adaptor protein 3	203320_AT	3.484	4.115	Modulator of receptor tyrosine kinases
TRIB3	Tribbles homolog 3 (Drosophila)	218145_AT	2.008	2.096	Negative feedback regulator of stress-regulated genes and Akt
	**Transcription factors**				
CCDC85B	Coiled-coil domain containing 85B	204610_S_AT	3.436	6.410	p53-induced
ELK3	ELK3, ETS-domain protein (SRF accessory protein 2)	221773_AT	3.497	2.874	Hypoxia, p53-regulated
KLF4	Kruppel-like factor 4 (gut klf)	221841_S_AT	3.185	2.110	
NR3C1	Nuclear receptor subfamily 3, group C, member 1 (glucocorticoid receptor)	211671_S_AT	4.386	2.841	
RXRA	Retinoid X receptor, alpha	202426_S_AT	2.445	5.405	Partner of retinoic acid and thyroid receptors
SALL1	Sal-like 1 (Drosophila)	206893_AT	4.854	2.299	
	**Growth factors and cytokines**				
BDNF	Brain-derived neurotrophic factor	206382_S_AT	2.083	2.075	Growth factor, anti-apoptosis
SLPI	Secretory leukocyte peptidase inhibitor	203021_AT	11.779	9.524	Protease inhibitor, growth factor
TNFSF9	Tumor necrosis factor (ligand) superfamily, member 9	206907_AT	2.083	4.219	Cytokine
	**Receptors and membrane-associated proteins**				

C10orf38	Chromosome 10 open reading frame 38	212771_AT	2.439	2.421	Putative membrane-associated
CXCR4	Chemokine (C-X-C motif) receptor 4	217028_AT	3.876	2.762	Receptor for SDF-1, HIV-1 co-receptor
LAPTM5	Lysosomal associated multispanning membrane protein 5	201721_S_AT	5.917	24.331	Membrane-associated
LAT2	Linker for activation of T cells family, member 2	221581_S_AT	3.968	6.369	Membrane-associated
MEGF9	Multiple EGF-like-domains 9	212830_AT	2.024	5.405	Putative receptor
NETO2	Neuropilin (NRP) and tolloid (TLL)-like 2	218888_S_AT	6.757	4.444	Predicted trans-membrane protein
PTCH1	Patched homolog (Drosophila)	209815_AT	2.242	4.310	Receptor for Sonic Hedgehog
SIGLEC15	Sialic acid binding Ig-like lectin 15	215856_AT	3.413	2.1786	Receptor for sialylated glycans
C9orf167	Chromosome 9 open reading frame 167	219620_X_AT	2.273	4.5045	Putative membrane-associated
SH3BP4	SH3-domain binding protein 4	222258_S_AT	2.004	2.0202	Membrane-localized, internalization of transferrin receptors
SMAP1	Stromal membrane-associated protein 1	218137_S_AT	2.571	2.8090	Clathrin-mediated endocytosis
		**Other**			
FAM129A	Family with sequence similarity 129, member A	217967_S_AT	7.874	3.0488	
HPCAL1	Hippocalcin-like 1	212552_AT	5.405	2.1739	Ca^{++} binding, neuronal signaling
MT1H	Metallothionein 1H	206461_X_AT	4.386	2.1598	Metal ion binding
MT1X	Metallothionein 1X	204326_X_AT	2.849	2.8011	Metal ion binding
MT2A	Metallothionein 2A	212185_X_AT	5.236	3.0864	Metal ion binding
PLAU	Plasminogen activator, urokinase	205479_S_AT	2.577	3.5336	Serine protease
TBC1D2	TBC1 domain family, member 2	222173_S_AT	2.488	2.8986	Rab GTPase activator
TGM2	Transglutaminase 2 (C polypeptide, protein-glutamine-gamma-glutamyltransferase)	201042_AT	3.086	5.5249	Protein cross-linking
TREX1	Three prime repair exonuclease 1	205875_S_AT	2.062	2.2026	DNA polymerase proof-reading function
